# The Practitioners' Bookshelf

**Published:** 1892-11-19

**Authors:** 


### THE PRACTITIONERS' BOOKSHELF.

### PICTORIAL MEDICINE.*

The article "Scrofula," in Part I. of the second volume of Dr. Byrom Bramwell's "Atlas of Clinical Medicine," accompanied, as we are now accustomed to expect, by its appropriate and admirable coloured plates, is very suggestive reading; more suggestive, perhaps, than Dr. Byron Bramwell himself appreciates. Dr. Bramwell discusses the nature and origin of scrofula, and he presents us also with Mr. Frederick Treves' views on the same subject at considerable length. What scrofula actually and essentially is neither of these learned and careful authors is quite prepared to tell us. But in one thing they are both agreed, that scrofula is by far the most common among those unfortunate human beings who are born of poor parents, reared in an impure atmosphere, poorly fed, and generally hampered and harassed as to their physical conditions by an unhappy and health-depressing start and environment. Tissue o a comp aratively embryonic character is probably the most marked feature of the glands of scrofulous persons. Their glands are ill-developed and poorly nourished—ill-developed because poorly nourished; and as the glandular structures are, of all the parts of the body, the least differentiated and the most embryonic, so are they the subjects of inflammatory action and disintegrating change on the smallest amount of provocation. The Prophet Jonah's gourd, which grew up in a night, withered also in a night; and this circumstance is illustrative of all organic structures of every kind. Do they grow quickly, and is the quantity and quality of their nutrition poor? Then it is certain that the rate and progress of their disintegration will be in strict proportion to the rate of their growth, and the poverty of the nutrition of which they have been the subjects. This puts us upon another question—upon two, indeed. What, in the first place, is the obvious suggestion for treatment; and what is the equally obvious suggestion for the gradual rooting out of scrofula and its congeners from the world? Dr. Byrom Bramwell furnishes us with pictures which are carefully drawn and admirably coloured; and we have heard doctors say of such pictures that they are "beautiful." They are dreadful, horrible! A science that looks far ahead anticipates the day when there will be no living subjects from which to draw and colour such pictures. The treatment, meantime, consists of years of intelligent "constitution building," if it is to be adequate; and, as for prevention, that is a vision to the effectual realisation of which medical scientists, who look far ahead, must devote all that portion of their lives which they can spare for the unpaid service of their fellow men. A worthy medical science can never be content until it cures all the diseases that are curable, and prevents all that are preventable.

Dr. Bramwell's "Atlas" continues to maintain the high level at which it started. It would be a pity if it should be otherwise. It is a monumental work, and promises to be well worthy of the century which proudly calls itself the "nineteenth," and which, so far at any rate as medicine is concerned, is justified in its honourable pride.

* "An Atlas of Clinical Medicine." By Byrom Bramwell, M.D., F.R.S., &c. (Edinburgh: J. and A. Constable, University Press.

